# Antidepressant Use and Its Association with 28-Day Mortality in Inpatients with SARS-CoV-2: Support for the FIASMA Model against COVID-19

**DOI:** 10.1192/j.eurpsy.2023.317

**Published:** 2023-07-19

**Authors:** N. Hoertel, M. Sanchez-Rico, J. Kornhuber, E. Gulbins, A. Reiersen, E. Lenze, B. A. Fritz, F. Jalali, E. Mills, C. Cougoule, A. Carpinteiro, C. Mühle, K. A. Becker-Flegler, D. R. Boulware, C. Blanco, J. M. Alvarado, N. Strub-Wourgaft, C. Lemogne, F. Limosin

**Affiliations:** 1 Université Paris Cité; 2 AP-HP, Paris, France; 3 University Hospital, Friedrich-Alexander-University of Erlangen-Nuremberg, Erlangen; 4 Institute for Molecular Biology, Essen, Germany; 5 Washington University School of Medicine; 6 Washington University School of Medicine, St. Louis, MO; 7 Saddleback Medical Group, Laguna Hills, CA, United States; 8 McMaster University, Hamilton, ON, Canada; 9 Université de Toulouse, Toulouse, France; 10 University Hospital Essen, Essen; 11 University Hospital, Friedrich-Alexander-University of Erlangen-Nuremberg, Erlangen, Germany; 12 University of Minnesota, Minneapolis, MN; 13 NIDA, Bethesda, United States; 14 Universidad Complutense de Madrid, Madrid, Spain; 15 DNDi, Geneva, Switzerland

## Abstract

**Introduction:**

To reduce Coronavirus Disease 2019 (COVID-19)-related mortality and morbidity, widely available oral COVID-19 treatments are urgently needed. Certain antidepressants, such as fluvoxamine or fluoxetine, may be beneficial against COVID-19.

**Objectives:**

The main objective was two-fold: (i) to test the hypothesis that the prevalence of antidepressant use in patients hospitalized with COVID-19 would be lower than in patients with similar characteristics hospitalized without COVID-19, and (ii) to examine, among patients hospitalized with COVID-19, whether antidepressant use is associated with reduced 28-day mortality. Our secondary aim was to examine whether this potential association could only concern specific antidepressant classes or molecules, is dose-dependent, and/or only observed beyond a certain dose threshold.

**Methods:**

We included 388,945 adult inpatients who tested positive for SARS-CoV-2 at 36 AP–HP (Assistance Publique–Hôpitaux de Paris) hospitals from 2 May 2020 to 2 November 2021. We compared the prevalence of antidepressant use at admission in a 1:1 ratio matched analytic sample with and without COVID-19 (N = 82,586), and assessed its association with 28-day all-cause mortality in a 1:1 ratio matched analytic sample of COVID-19 inpatients with and without antidepressant use at admission (N = 1482) (Figure 1).

**Results:**

Antidepressant use was significantly less prevalent in inpatients with COVID-19 than in a matched control group of inpatients without COVID-19 (1.9% versus 4.8%; Odds Ratio (OR) = 0.38; 95%CI = 0.35–0.41, p < 0.001) (Figure 2). Antidepressant use was significantly associated with reduced 28-day mortality among COVID-19 inpatients (12.8% versus 21.2%; OR = 0.55; 95%CI = 0.41–0.72, p < 0.001), particularly at daily doses of at least 40 mg fluoxetine equivalents (Figure 3). Antidepressants with high FIASMA (Functional Inhibitors of Acid Sphingomyelinase) activity seem to drive both associations.

**Image:**

**Figure:**
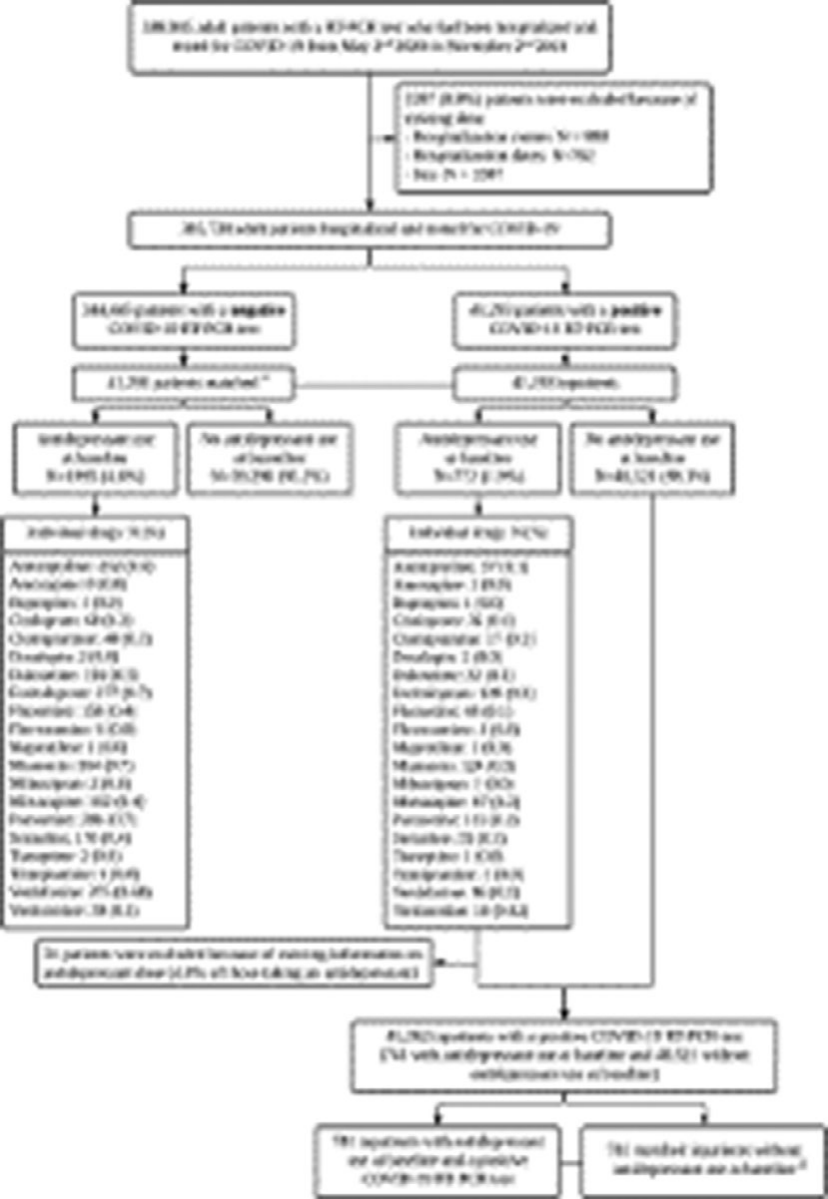

**Image 2:**

**Figure:**
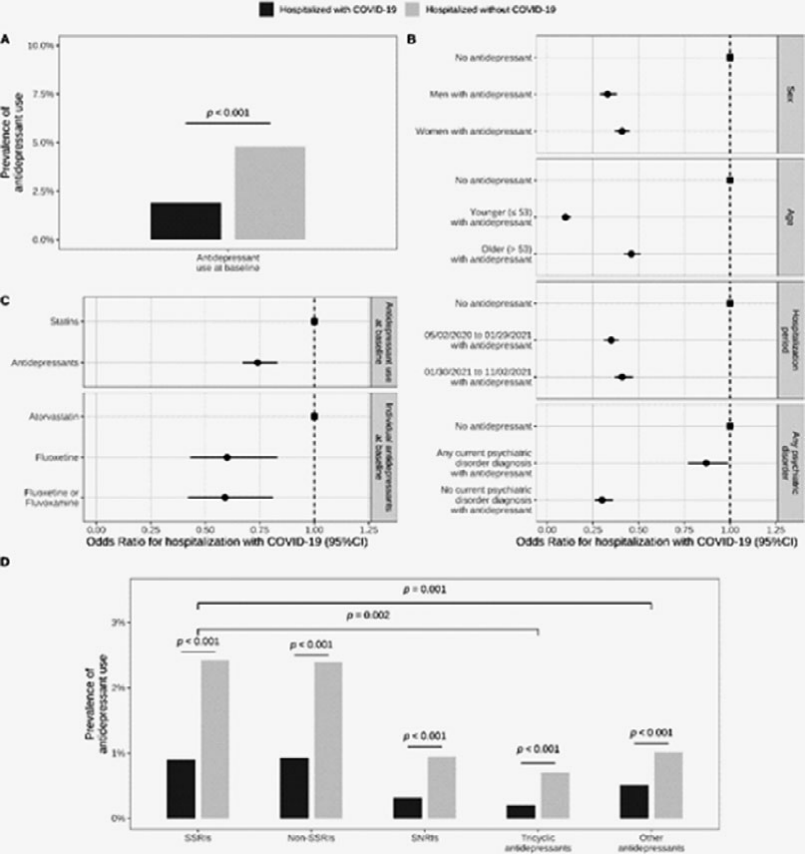

**Image 3:**

**Figure:**
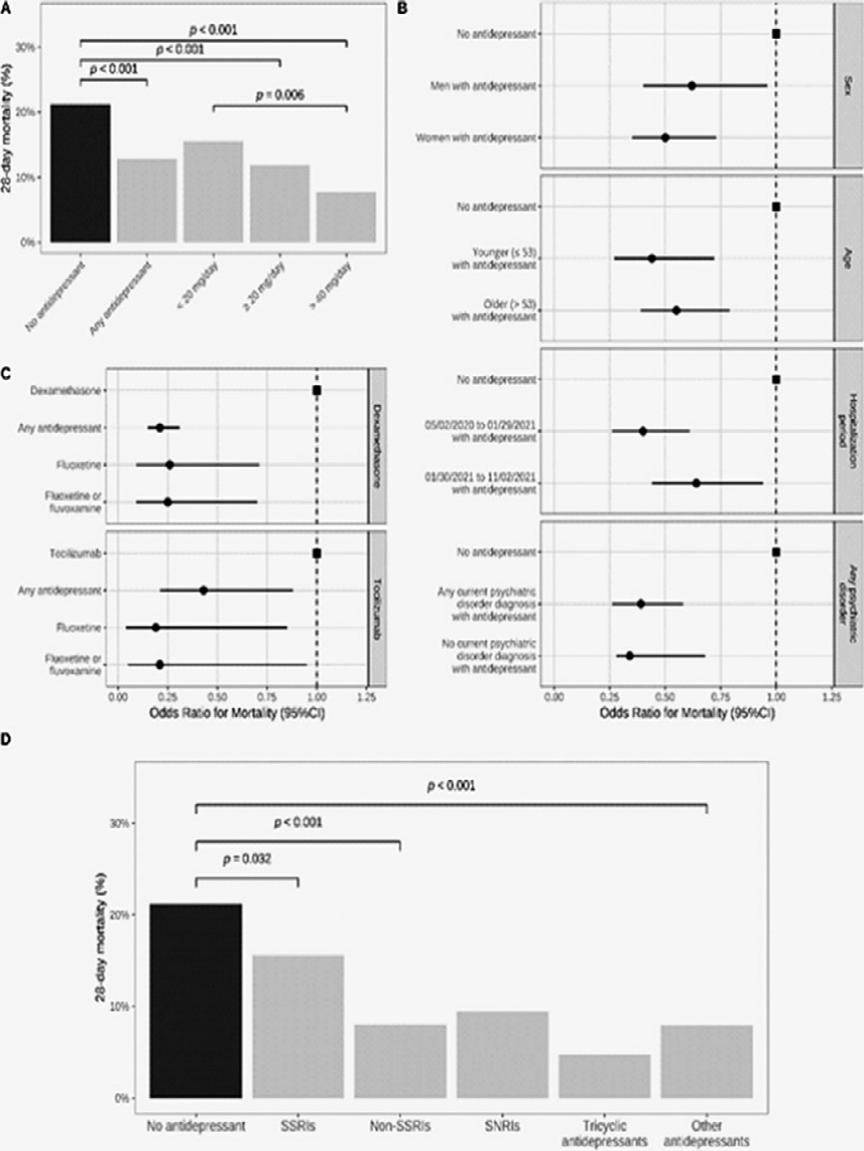

**Conclusions:**

Antidepressant use is associated with a reduced likelihood of hospitalization in patients infected with SARS-CoV-2 and with a reduced risk of death in patients hospitalized with COVID-19. These associations were stronger for molecules with high FIASMA activity. These findings posit that prospective interventional studies of antidepressants with the highest FIASMA activity may be appropriate to help identify variant-agnostic, affordable, and scalable interventions for outpatient and inpatient therapy of COVID-19.

**Disclosure of Interest:**

None Declared

